# Mr. Hill, on Opium

**Published:** 1803-12-01

**Authors:** G. Nesse Hill

**Affiliations:** Chester


### To Dr. BRADLEY.

Sir,

If you and your Readers are not weary of the subject, I have now transmitted for insertion in the Journal a few further Remarks on the Doctrines advanced by Mr. Ward, respecting the modus operandi of opium. This writer, Vol. vi. p. 480 of the Medical Journal, says, "Opium joined with other antispasmodics and tonics;" a few lines lower down of the same page, he declares his belief that opium acts more directly and simply as a sedative when applied externally than when given internally. Here then is an inference that its action is in both modes sedative, but more directly so outwardly than inwardly. Now I cannot conceive how it is possible to reason fairly in this manner, seeing that whatever is its actual power (and we know it to be very great) one way or the other, it certainly will exert that power more quickly and forcibly on such an organ as the stomach, than on any part of the whole external surface that can be mentioned; that it may act more simply ab externo will be readily allowed, but more directly is quite another thing; the cases Mr. W. has so clearly communicated, where opium was externally applied, are exactly so many many proofs of his using it as a tonic, unless he will say they were all sthenic diseases, which I am confident he will not.

At p. 441, Medical Journal for July, 1799, the case of G. P. is related : A typhus, in which Mr. W. exhibited five grains and a half of opium to a subject fourteen years of age in forty-eight hours. Did he believe he was administering a medicine calculated to diminish the tone of the system, or a sedative? The effects he has recorded are an answer, for if any share of the success arose from opium, it was evidenced by abatement of delirium, tranquil sleep, reduction of fever, return of appetite, and lastly his pulse, which had hitherto varied from 100 to 120, was now only 80, but *stronger*. Were all these valuable changes wrought by any substances acting as sedatives, or medicines which "directly and powerfully" diminish the powers of the body? The remainder of the narrative contains farther proofs of the tonic action of opium internally and externally.

The succeeding cases are by Dr. Percival, both of which are additional proofs of the force of truth; it cannot for an instant be supposed that a practitioner of Dr. P's allowed skill and accurate observation, would have directed the use of laudanum as a sedative, or diminisher of the powers of the human system, to a female forty-six years of age, labouring under epilepsy, anomalous gout, and temporary insanity. The same observation applies to his second case, of a gentleman of delicate make, ill of low fever, with delirium and a languid pulse; these symptoms were removed by the use of opium, externally applied and internally taken, but not I presume from its lessening the tone or vigor of the body; but this truly eminent physician shall give us his own words on this important matter. Describing the case of a debilitated epileptic young woman, in his Essay on Blisters, p. 228, he says, "She was seized with convulsions of the epileptic kind, and had six fits successively; she was cold, feeble and languid, and complained much of sickness and pain in her head." The following medicines were prescribed. ℞. Tinct. valer. vol. fuliginis a. ℨß. laud. liq. gutt. xl. M. cap. coch. parv ij. omn. hor. in aq. spiritusque viij. gallici. Next day this patient got free of her fits and was better every way; not I presume from the debilitating effects of ten drops of laudanum every hour, but by the tonic property of this invaluable remedy conjunctly with the stimulants. Farther, Dr. P. in his cases of dropsy, vol. 2, of the same work, p. 157, says, "I found her quite exhausted with incessant retchings; her pulse was so feeble as to be scarcely perceptible, her legs and thighs were affected with a most painful spasm; gentle cordials were directed to support the patient's strength, warm fomentations were applied to her legs and thighs, and an opiate was administered." The result of this judicious treatment was immediately some hours sleep and cessation of the vomiting. Will any advocate for the sedative action of opium, affirm that these salutary results arose from sedative remedies, or those which diminish animal energy and weaken the powers of life? Would Dr. P. have been justified in using opium in this case of extreme debility, if he had not known, from long experience, its excellent faculty of restoring the weakened energy of the frame, the most safely and speedily of any medicine within his knowledge or reach? I cannot take leave of this author without citing two more instances from his useful Essays, in confirmation of this point; these will be found at page 208 and 209, vol. ii.; the one a confluent small-pox, accompanied with convulsions and extreme debility, both relieved by opium in clysters and by the mouth; the other, a lady of a delicate habit, much enfeebled by scurvy, hysteria, anorexia, delirium, convulsions, &c. with a quick, fluttering, irregular pulse; a grain of opium every night with other tonics and stimulants proved curative. Opium is an agent of the most powerful kind; if this power over the functions of the human body were of the depressing, debilitating, sedative kind, which Mr. W. believes it to be, I am afraid Dr. P. would have had but a small chance of being serviceable to his patient. Before quitting this part of the subject it will not be inappropriate to observe, that although Dr. Cullen, after, as Mr. W. says, "a close investigation," places them, i. e. wine and spirits, among the narcotic sedatives or opiates, and says, that at first they both banish languor and promote cheerfulness if the quantity be gradually increased; yet, "at length the sedative power entirely prevailing, the animal functions both of sense and motion are gradually weakened, &c." Here we have one of our greatest writers on the Materia Medica arguing for a powerful medicine containing two opposite qualities, whereas it is well known that no man depended more upon the tonic powers of opium in his whole First Lines of the Practice of Physic, nor has any practitioner given more cautions against its administration in sthenic diathesis, where sedatives are so eminently serviceable; but it is curious enough to observe how often theory and practice are totally at variance in the works of many justly esteemed medical authors. As proofs proofs of what is here advanced, the reader will doubtless recollect sufficient instances in Dr. Cullen's valuable work just mentioned; but lest he should not, I presume to quote his treatment of Rheumatism. Paragraph 468, "Opiates, except when they are directed to procure sweat, always prove hurtful in every stage of this disease." Now, if opium be a sedative, or medicinal agent, which when introduced into the human body diminishes power or lessens sthenic diathesis, why does Dr. C. not give it rank amongst all those agents which he has enumerated as curative of a sthenic, or as he terms it, an acute disease? He would certainly have done so, had he not practically known that opium was a tonic stimulant. The whole tenor of his great work may be adduced in proof of this fact; he always directs its use in cases of direct and indirect debility, and condemns it in the opposite states. Turn to Par. 570, "In the painful and restless nights of the gout, opiates may be given with safety and advantage, especially in the case of persons advanced in life and who have been often affected with the disease." Shall proofs be multiplied, go on to Par. 580, and we find opium the author's sheet anchor, when this highly asthenic disease has attacked the stomach.

Par. 620, on Small-pox; 802, on Hæmorrhagies; 923, on Phthisis Pulmonalis, where we find that opium "certainly increases the phlogistic (or sthenic) diathesis of the system, but commonly it does not so much harm in this way, as it does service by quieting the cough and giving sleep," i. e. by removing debility pro tempore, cessation from coughing follows and sleep succeeds of course. Farther, "Opiates are supposed to be hurtful by checking expectoration; but they do it for a short time only, and after a sound sleep, the expectoration is more easy in the morning than usual." Now, unless a man be so impregnably shut up in theory as to be invulnerable, he will never call Dr. C's system in aid of proof of the sedative properties of opium; but as this author ranks deservedly high in the public estimation, and must be looked up to as an authority of great weight, I shall therefore recall to the observation of those who choose to pursue the subject what he says at Par. 1014, when treating of difficult menstruation: "We impute this disorder partly to some weaker action of the vessels of the uterus, and partly perhaps more especially to a spasm of its extreme vessels." "We have commonly found the disease relieved by employing some of the remedies of suppression, (i. e. tonics, vide Par. 1004, 1006) immediately before the approach of the period, and

 at at the same time employing opiates." But Mr. W. says, opiates diminish energy, lessen action, and weaken the system.

How came Dr. C's practice in Dysmenorrhœa to be successful? Or it may with no less propriety be asked, how he came to forbid opium in the early stages of catarrh if it be a sedative, whilst he recommends sedative remedies of far less power? No, he directs its use in the advanced state of this disease, when debility has supervened, vide Par. 1066. The reader may afterwards turn his eye to the diseases termed dyspepsia, hypochondriasis, epilepsy, &c. all of which admit the same remarks. In Par. 1342, we have the whole question settled thus, "When the disease (epilepsy) depends upon a plethoric state, in which bleeding (a sedative remedy) may be necessary, the employment of opium (a tonic remedy) is likely to be very hurtful; but when there is no plethoric or inflammatory state present, (sthenic diathesis) and the disease seems to depend upon irritation or upon increased irritability (asthenic diathesis) opium is likely to prove the most certain remedy." Or, in the same way, at Par. 1463, cholera is directed to be thus managed, "Or when a dangerous debility seems to be induced, the irritation (arising from that dangerous debility) is to be immediately obviated by opiates in sufficiently large doses, but in small bulk, and given either by the mouth or by glyster;" and it is thus we are directed to act with a medicine which Mr. W. has taken no small pains to prove is one of the most powerful debilitants in the circle of the Materia Medica. In short, as I have already observed, we might thus traverse the whole of the First Lines and make similar remarks. What have been adduced are sufficient; it is time to return to the Observations, where we shall in vain look for any reasoning which will overturn the Cullenian practice; on the contrary, the very next instance in Mr. W's first paper, p. 445, is corroborative of the justness of Dr. Cullen's practical remarks, illustrated by a case of chronic rheumatism of "seven months continuance," which fell under Mr. W's care: Ease and rest were only to be procured by opium; when it was combined with sedatives or debilitants its effects were destroyed." And no wonder they should; when its effects are diametrically opposite, how could they be expected to act conjunctively? And who anticipates a beneficial result from aperients (sedatives) in chronic rheumatism? I sincerely hope Mr. W. will never suffer one hour's pain of the same kind I have endured from this miserable disease; if however he should be be so unfortunate, I can assure him he will be thankful there are such medicines as tonics, and he will gratefully acknowledge that opium deserves to stand at the very head of the number. The inferences from this case are all convincing arguments of the tonic powers of the remedy, if the disease to which it was so assiduously applied be allowed to rank with the asthenic class, which I presume no practitioner will controvert; as to the difference of its effects when internally and externally applied, Mr. Ward's observations afford opportunities of speaking more at large hereafter.

Humanity shudders at the bare mention of Hydrophobia. Should the observer's suggestions on this head be adopted, and crowned with success, he will have acquired a never-fading laurel; although I ardently pray this may be the case, I would not have this honourable badge sullied by a misconception of the true nature and wonderful powers of the agent by which such fame is obtained, which will most certainly be the result, if he persists in calling opium a sedative and hydrophobia an asthenic disease. The truth of the latter assertion is as clear in my estimation as that tetanus belongs to this class, and that if opium be able to cure them it must be from its highly tonic properties; that it will effect this most desirable of events is well known by experience as to tetanus; I trust an occasion will be long wanting to both of us to put it to the full test in hydrophobia. In the other parts of the treatment of this disease, as recommended by Mr. W. he has inserted two lines of some importance to my views, viz. "to support the strength, glysters consisting of good broth, or milk with thirty or forty drops of laudanum in each, p. 448; no animadversion is necessary.

Vol. vi. p. 6, of the Med. and Phys. Journal, affords a continuation of the observations as to the external use of opium. The case of J. Jackson, a debilitated maniac with a fractured leg, is the only case recorded in this paper; but it is an additional one to prove the tonic powers of the remedy, if a feeble pulse of 120 in a man of fifty-three years of age, incessant muttering, universal tremulous, tongue and gums furred with brown streaks, and continued watchfulness, are so many proofs of asthenic insanity. That I have always found them such during an extensive practice in this department I may safely aver, and that opium with other tonics, under due regulation, have always proved curative; nor do I hesitate in saying, that if as large a dose of opium had been given internally to Jackson, as

Mr.

Mr. W. we shall hereafter find, exhibited to some persons by way of experiment, merely to endeavour to prove its sedative action, he would have reaped every desirable benefit, and been as tranquil as he was the morning after the friction, which we are told had the effect of reducing the quickness of the pulse, and reducing it just so much nearer the healthy standard, as the medicine proportionally took off debility by communicating its tonic energy. The few drops given in a draught (five every four hours) were not equal to the production of any such effect; but when we have such clear evidence of the salutary effects of half a drachm of opium introduced by friction into the system of a maniac, it cannot be otherwise considered than as a great acquisition to the modus curandi. There are other points in this case evidencing the tonic action of opium, besides returning reason and slowness of the pulse, such as increase of appetite, notwithstanding Mr. W. contends farther on, that it always diminishes the appetite when taken internally. But here is a case proving the contrary; far on in the treatment the sufferer took two grains every night, which, as before remarked, had he done at the first, the same beneficial effects had followed; that it must have acted as a tonic, when externally used, and not as a sedative, is manifest, if friction itself be a tonic stimulant, and quicker absorption the consequence; but if the reverse be the fact, that is, friction produces torpor, and opium when thereby applied to the lymphatics diminishes their action, then indeed it is a sedative, and every thing must be conceded to Mr. Ward's reasoning.

Vol. vi. p. 387, affords a case successfully treated by opiate frictions under the care of Mr. White, of Bath, which perfectly corresponds with all I have hitherto seen in confirming the tonic powers of opium. Mr. White's patient laboured under a number of symptoms originating in debility, evidenced by the quickness and feebleness of the pulse, all the distress was apparently removed by the rubbing in of ℥ß tinct. opii on the abdominal surface. Mr. White gives no opinion on the modus operandi of the opium, but concludes his narrative thus: "Her pulse was reduced to 96 on the third day, which before was exceedingly quick;" that it was feeble had been before remarked, so that its reduction nearer to the standard of health, and proportionate removal of feebleness, are effects attributed with every other success to the opium; of consequence, if this be true, the stimulus of the friction with the tonic power of the remedy restored the patient.

In the same volume, likewise, at p. 431, and sequel, will be noted a continuation of the subject by Mr. Ward. Two cases are there recited; the first, by Mr. Jenkinson, is one where great prostration of strength was the cause of all the mischief; but not being informed what quantity of opium was exhibited in any way, no opinion can be given, whether it was merely sufficient to remove the direct debility, or whether in such large doses as to superinduce the indirect. From what is said however, it may safely be inferred, that the good effects arising from the remedy in this poor woman were owing to its tonic power; for Mr. J. says, "Her life was thereby certainly rendered more comfortable, and her existence prolonged several days." No sedative power could be applied but must have been productive of opposite consequences; of this truth I am as fully persuaded as of any one acknowledged not to admit of doubt.

Mr. Boutflower's tetanic case comes next, and from which nothing can be deduced in the remotest degree but what tends to confirm the general opinion of the asthenic nature of the disease. That it was removed by opium is not less apparent, and that it would have been so removed by its internal exhibition is equally certain; for it is observable, Mr. B. commenced its exhibition in doses of one grain every six hours, which would probably have been too large a quantity for his young patient under any other circumstances; but in the outset of this hourly encroaching disorder it certainly was too small, for he subsequently bore with advantage two grains every night besides ʒij tinct. opii by friction every twelve hours. Will it be said by any medical artist, that when the external application was left off, if a proportionate increase had been made in the internal, a cure would not have been effected? Let it not be thought that by this or any similar remark, I would presume to call in question, or doubt the salutary effects of opiate friction; so far from it, I highly esteem the improvement, inasmuch as it enlarges the bounds of utility of this noble remedy; I only wish to be understood to firmly believe in the uniformity of its action and effects, however applied, and that it is always capable of increasing the tone or vigour of the system. The whole of what has been said on the last case is confirmed, if confirmation was wanting, by the practice of M. Mursuina, of Berlin, an outline of which we have at page 280 of this volume. At page 481, the case of Jackson is again brought forward; in addition to to what I have already said, I may be allowed to observe, that if this man had been bled, purged, and undergone the too common routine of debilitating practice, I believe the sheers sheers of Atropos would speedily have performed their office; or had he escaped such a termination of his complaints, confirmed insanity or perpetual idiotism had been his lot. The rest of the cases inserted in this paper all tend to confirm the tonic powers of opium; the one immediately succeeding Jackson's was a female patient, " sinking fast, her pulse very rapid and feeble, and she had a tendency to delirium." Farther, " Wine, which in this state seemed highly requisite," (as a stimulant I presume, and of course temporarily to remove debility, for this must be the idea of all who believe in its stimulant powers as they are termed) " but she refused to take it; its place therefore was supplied"—by what? Why, Mr. Ward says, by a most powerful sedative, and the patient's " pulse and intensity of heat were gradually reduced, the diarrhœa was stopped," and I presume her fast sinking prevented; not by a remedy which produces torpor, diminishes vital energy, destroys power, &c. the uniform effects of sedatives, but by one which acts diametrically opposite. But lo! the medicine, which a page back ranked with the tonics, is now, in the inference drawn from Baylis's case, become a powerful sedative, owing to which property it was found to " diminish irritability, sensibility, and mobility of the system." Nothing is said of the debility which was the foundation of the whole mischief, and which the highest known tonic stimulant alone could remove from its very base, consequently all the edifice of symptoms must give way, and finally also be removed; indeed, this case is a striking illustration of the truth, that opium is not in any other sense a sedative.

Mr. Ward mentions, with strong emphasis, the effects of the remedy on the pulse, viz. That whatever was its alteration in any other respect, its " frequency was diminished." I ask, can there be a more prominently striking proof of the invigorating agency of the medicine? for unless Mr. Ward means to prove that its frequency being diminished, its force became weaker, and that this increased weakness was useful and proper, he can, in my humble opinion, prove nothing at all, but what must strongly tend to determine, in the most direct manner, the question at issue against his theory.

*Chester, Oct.* 8, 1803. G. NESSE HILL.

CASE

